# Economic drivers and systemic disparities in invasive coronary angiography: Insights from a national claims database

**DOI:** 10.1371/journal.pone.0356496

**Published:** 2026-09-03

**Authors:** Seyed Sahab Aarabi, Farbod Semnani, Kiavash Semnani, Shirin Esmaeili, Rajabali Daroudi, Mehdi Rezaei, Mohammad Effatpanah, Mohamad Mehdi Nasehi, Majid Haghjoo

**Affiliations:** 1 National Center for Health Insurance Research, Tehran, Iran; 2 School of Medicine, Tehran University of Medical Sciences, Tehran, Iran; 3 Department of Health Management, Policy and Economics, School of Public Health, Tehran University of Medical Sciences, Tehran, Iran; 4 Department of Orthopedics, School of Medicine, Tehran University of Medical Sciences, Tehran, Iran; 5 Pediatric Psychiatry Department, School of Medicine, Imam Khomeini Hospital, Tehran, Iran; 6 Pediatric Neurology Department, Mofid Children’s Hospital, Shahid Beheshti University of Medical Sciences, Tehran, Iran; 7 Cardiac Electrophysiology Research Center, Rajaie Cardiovascular Institute, Tehran, Iran; Iranshahr University of Medical Sciences, IRAN, ISLAMIC REPUBLIC OF

## Abstract

**Background:**

Despite the prominent economic burden of coronary care, detailed analyses of factors associated with expenditure in developing nations remain sparse.

**Objectives:**

This study aimed to provide such analyses using data from a national cohort of patients undergoing invasive coronary angiography (ICA).

**Methods:**

Patient data and billable services were retrieved from the Iran Health Insurance Organization (IHIO) database. Data from 158584 hospitalizations were analyzed. A multi-pollutant index was constructed using weighted quantile sum regression. Hospital case volume and provincial ICA rates were modeled using generalized linear models. Three-tier (patient, hospital, and province) generalized linear mixed models were devised for inpatient costs, revascularization odds, and length of stay (LOS).

**Results:**

17.0% of episodes pertained to acute coronary syndrome. Median inpatient cost was PPP$2033. Interventional services accounted for the largest share of costs (37.1%). Out-of-pocket expenditure was 16.6%. Revascularization was performed in 47.2% of hospitalizations. Ambient pollution, lower socioeconomic deprivation, and higher capacity (active beds and angiography devices) were associated with provincial ICA rates. Male sex and older patients had higher revascularization odds, prolonged stays, and incurred higher costs. Procedure complexity (Cost Ratio: 4.37; 95% CI 4.34–4.39) was a major predictor of costs, along with private ownership, heart center status, and weekend hospitalization. Non-cardiac hospitalizations had prolonged LOS. Less deprived provinces had higher revascularization odds for chronic indications.

**Conclusions:**

ICA utilization, outcomes, and costs in Iran were found to be significantly associated with clinical complexity, institutional characteristics, regional capacity, and environmental exposures. These findings are critical for identifying future funding and research priorities.

## 1. Introduction

Cardiovascular disease (CVD) is the leading cause of morbidity and mortality worldwide [1,2]. Ischemic heart disease (IHD) is the most prominent category– being responsible for over 44% (8.91 million) and 46% (193 million) of the mortality and disability-adjusted life years (DALYs) attributable to CVD, respectively [2]. Despite progress in mitigation of IHD [1], population aging and inclines in metabolic risk factors have led to an overall increase in the burden of disease [2,3]. The increase is expected to continue, with a global increase of 80% and 62% in IHD mortality and DALYs in future decades [3]. Accordingly, the economic burden of IHD is a major contributor to overall costs (e.g., 11% of total healthcare costs in the EU), and a critical indicator of the quality of care [4]. These observations necessitate continued assessment of healthcare systems’ capacity and performance regarding IHD care [2,4].

Aggregate annual costs of IHD in the US and EU have been estimated to exceed $260 and €77 billion, respectively [4,5]. Direct healthcare expenditure contributes to more than half and a third of the overall costs in these regions [4,5]. Healthcare costs are also expected to be responsible for a major portion of the projected increases in IHD costs [5]. Inpatient care is the most prominent contributor to the direct healthcare costs (79% in the EU) of IHD [4]. Invasive coronary angiography (ICA) and revascularization, in turn, serve as both major contributors to costs, and key indicators of access to coronary care [6,7]. However, estimates from developing nations are sparse and less reliable [8–10].

Available estimates indicate that the growth in IHD burden more prominently affects countries with low to middle sociodemographic development [3]. This holds true for countries in the Middle East region, where persistent challenges in primordial and primary prevention have led to a comparatively high burden of disease despite earlier successes [9,11]. Among Middle Eastern nations, Iran maintains the highest prevalence and incidence of IHD, while it has succeeded in attenuating the subsequent burden of disease [11]. With this, demographic shifts, and more recent transformations in the national health system – driven by budgetary restraints due to geopolitical developments – have led to a critical need for reassessment of the drivers of costs and funding priorities [12,13]. Analyses of expenditures on secondary and tertiary care are necessary to maintain and improve the health system’s performance, alongside a focus on disease prevention.

Previous estimates from Iran are mostly reliant on data from few hospitalization in local or regional care centers [12,14]. Most studies also focus on providing estimates for the overall economic burden of IHD, with minimal exploration of factors associated with higher expenditure – particularly regarding key services such as ICA and revascularization [12,14,15]. The current study aims to provide timely estimates of drivers of inpatient costs; service uptake; and systemic factors associated with disparities in the economic burden of coronary care by leveraging data from a large-scale national cohort of patients undergoing ICA.

## 2. Methods

### 2.1. Study design and data sources

We conducted a nationwide, retrospective cohort study using administrative claims data from the Iran Health Insurance Organization (IHIO) database. The study period spanned one solar calendar year (March 21, 2023 to March 19, 2024). IHIO covers more than 45 million Iranians [16]. The data used in this study were accessed for research purposes from 30/08/2025.

The coverage is subdivided to multiple insurance funds: the Rural Fund provides health coverage to residents of rural areas and small towns; the Government Employees Fund is a contributory insurance scheme designed for civil servants (active and retired); the Iranians Fund is targeted at urban residents not covered by any other formal insurance; and the Other Social Groups Fund serves as a catch-all category for specific groups designated as high-priority or vulnerable [17]. This study adheres to the Strengthening the Reporting of Observational Studies in Epidemiology (STROBE) reporting guidelines for observational studies.

### 2.2. Study population and patient selection

The primary data was integrated from two IHIO registries: an Inpatient Admission Registry containing demographic data, administrative details, and clinical diagnoses coded using the International Classification of Diseases, 10th Revision (ICD-10); and a Service Claims Registry providing records of all billable services. The initial retrieval identified hospitalizations involving ICA. Patients younger than 18 years, and hospitalizations with unspecified or missing demographic data were excluded. Facilities with an annual ICA volume of less than 6 cases were excluded to minimize the impact of low-volume outliers on facility-level estimates. Costs were converted to International Dollars (ID) using the Purchasing Power Parity (PPP) conversion factor for Iran [18].

### 2.3. Variable definitions

Variables were categorized into a three-level structure:

- **Patient-level variables:** age; sex; insurance fund; clinical diagnoses – Acute Coronary Syndrome (ACS), Stable Ischemic Heart Disease (SIHD), etc., coded using the ICD-10 (S1 Table in S1 File); physician characteristics (specialty and rank); discharge status; and admission/discharge date.
- **Hospital-level variables:** ownership, capacity, and geographic status (province center vs. periphery). Facilities in the top quartile of ICA volume were deemed “high-volume”. Heart centers were also marked.
- **Province-level variables:** active bed capacity (per 1,000) and angiography device density (per 1,000,000) were retrieved from the annual report of the Treatment Deputy of the Ministry of Health and Medical Education [19]. Provincial socioeconomic deprivation was derived from a systematic review of development indices in Iran [20]. This rank-ordered index synthesizes findings from geographical analyses evaluating multiple deprivation domains, with heavy weightings on infrastructural deficits, housing, and employment. For this study, deprivation was treated as a rank-ordered variable where a higher rank indicates lower levels of deprivation.

We assessed angiographic services, revascularizations, and ancillary imaging services. Interventions were categorized as complex or simple. Multi-vessel/multi-stent single-vessel PCI, primary PCI, complex lesions (e.g., Chronic Total Occlusion (CTO) or unprotected left-main), and/or CABG procedures requiring four or more grafts were designated complex. These procedures were aggregated into a single complex variable for the hierarchical models to provide a robust estimate of the overall economic burden associated with high-intensity care. This approach was necessitated by the need for model stability and to account for clinical scenarios where patients required overlapping complex services during a single index hospitalization. Also, the procedural data, associated costs, and length of stay from staged revascularizations were merged into a single index record per patient to ensure independent observations for the predictive models.

### 2.4. Multi-pollutant exposure assessment

Meteorological (temperature, wind speed, atmospheric pressure, and precipitation) and air quality data (CO, NO2, SO2, O3, PM2.5 and PM10 concentrations) were retrieved by provinces [21,22]. Missing values were imputed using Multivariate Imputation by Chained Equations (MICE) [23,24]. We calculated a 3-day average lag for environmental variables to account for the physiological delay in acute cardiovascular response to environmental stressors [25,26]. Lagged exposures were mapped to individual hospitalization records based on the province of hospitalization and date of admission. This integrated environmental-clinical dataset was primarily utilized to assess the effect of air pollution on the volume of ACS related angiographies, facilitating the identification of dominant pollutants through mixture modeling and the construction of a composite multi-pollutant index for subsequent analyses. Rather than serving as a primary economic endpoint, the air pollution assessment was designed to capture external environmental pressures that influence provincial rates of acute coronary admissions.

### 2.5. Statistical analysis

#### 2.5.1. Descriptive and preliminary analyses.

Baseline characteristics of the cohort were reported using descriptive statistics. Data cleaning, management of high-dimensional registries, and handling of missing data were conducted using Python (version 3.12.12; pandas and numpy libraries).

#### 2.5.2. Multi-pollutant mixture modelling.

We employed weighted quantile sum (WQS) regression to estimate the joint effect of a pollutant mixture on ICA volume for acute indications [27]. This approach facilitates identification of high-contribution pollutants while addressing the inherent multi-collinearity of environmental data. The model was adjusted for meteorological covariates, province-level bed capacity, and month and day of the week. A composite WQS Index was constructed using weights from the ACS cohort. This index was integrated as a fixed effect in the hierarchical models.

#### 2.5.3. Hospital and province-level ICA volume and rate analysis.

The association between hospital-level characteristics and ICA volume was assessed using a Generalized Linear Model (GLM) with a negative binomial distribution to accommodate overdispersion in the count data [28,29]. We conducted univariate and multivariate (adjusting for facility ownership, bed capacity, and geographic status) analyses. Given the limited sample size (N = 31) on the province level, univariate GLM with a Gamma distribution and log link was utilized to model ICA rates (per 100,000) – providing an optimal fit for non-negative highly skewed rate data [30].

#### 2.5.4. Multilevel hierarchical modeling (GLMM).

We devised a three-tier (patient, hospital, and province) Generalized Linear Mixed Models (GLMM) to account for the nested structure of data. Three outcomes were evaluated: revascularization was modeled using a binomial distribution with a logit link to report Odds Ratios (OR); length of stay (LOS) and Hospitalization Costs were modeled using a log-normal distribution to report Rate Ratios (RR) and Cost Ratios (CR) respectively. Fatal hospitalizations were retained in all cost and LOS models to accurately reflect the total economic and resource burden from the payer's perspective. Patient, hospital, and province-level variables were entered as fixed effects, while hospital and province IDs were treated as random intercepts. Models were stratified by clinical indication (total, ACS, and SIHD).

#### 2.5.5. Model validation and software.

Model stability and fit were assessed using DHARMa residual diagnostics [31], including dispersion and outlier tests. Multi-collinearity was monitored using the Variance Inflation Factor (VIF) [32]. To quantify variance across levels, we reported Intraclass Correlation Coefficients (ICC), while model explanatory power was evaluated using Marginal and Conditional R^2^ [33]. Ninety-five percent confidence intervals for the ICCs were obtained by parametric bootstrap (1000 simulations from the final fitted model, each refitted to recompute the variance-partition coefficients), reported as the 2.5th and 97.5th percentiles of the bootstrap distribution. Statistical analyses and visualization were performed in R (v4.4.2) using the glmmTMB, gWQS, performance, DHARMa, ggplot2, and forestploter packages.

In summary, our statistical pipeline comprehensively evaluates economic and systemic drivers of coronary care. First, descriptive analyses established baseline demographic and clinical profiles and economic burdens. Second, multi-pollutant mixture modeling assessed environmental impacts on acute admission volumes. Third, generalized linear models evaluated how facility and provincial infrastructures drive regional angiography rates. Finally, three-tier generalized linear mixed models identified hierarchical predictors of revascularization, costs, and length of stay, successfully isolating systemic disparities from clinical complexity.

### 2.6. Ethical considerations

The study protocol was approved by the Ethics Committee of the School of Public Health, Tehran University of Medical Sciences (Approval ID: IR.TUMS.SPH.REC.1403.319; Approval Date: March 16, 2025). All patient identifiers were anonymized via hashed IDs prior to access. It was not appropriate or possible to involve patients or the public in the design, conduct, reporting, or dissemination plans of our research due to the retrospective nature of the study and the use of de-identified national administrative claims data. As a result, the research was classified as exempt from individual informed consent.

### 2.7. Use of Artificial Intelligence

During the research process, the authors utilized Gemini to assist in the technical optimization of the statistical scripts. Specifically, the technology was used for debugging and refining the GLMM and WQS regression code to ensure computational efficiency. All AI-generated code was manually reviewed for logical accuracy and validated against standard statistical outputs by the authors. Additionally, the AI was used for linguistic polishing to improve the clarity and flow of the scientific narrative. The authors reviewed the final output to ensure the absence of plagiarism and the accuracy of all clinical interpretations.

## 3. Results

### 3.1. Baseline cohort clinical characteristics

158584 hospitalizations were analyzed. The study population had a mean age of 63.1 years with male predominance (57.1%). The Rural Fund represented the largest group at 34.7%. SIHD was the primary diagnosis – accounting for 56.8% (n = 90,031) of the cohort. Hospitalizations predominantly occurred in governmental academic centers (67.2%) and were largely concentrated in province centers (84.0%). Median LOS was 1.74 days, with a 23.9% rate of same day discharge (Table 1). Significant temporal patterns were noted in clinical presentation (S1 Fig and S2 Table in S1 File). SIHD/ACS ratio was lowest on the weekend (Fridays), and reached a nadir at the end of the calendar year (March). Few patients (5.9%) had repeat ICA during the study period (S2 Fig in S1 File).

**Table 1:** Baseline characteristics of the invasive coronary angiography (ICA) hospitalization cohort.

Characteristic	Total (N = 158584)
***Demographic and Socioeconomic***	
**Age, years (mean ± SD)**	63.1 ± 11.0
**Age group, n (%)**	
18–39 years	2827 (1.78)
40–64 years	82213 (51.84)
>= 65 years	73544 (46.38)
**Sex, n (%)**	
Male	90539 (57.09)
Female	68045 (42.91)
**Insurance fund, n (%)**	
Rural fund	55099 (34.74)
Government employees fund	46854 (29.55)
Iranians fund	34759 (21.92)
Other social groups fund^*^	21872 (13.79)
***Province and Hospital***	
**Top 5 provinces by total number of ICA hospitalizations** ^**$**^ **, n (%)**	
Tehran	32181 (20.29)
Khorasan Razavi	19096 (12.04)
Fars	14229 (8.97)
Isfahan	9212 (5.81)
Mazandaran	9202 (5.80)
**Top 5 provinces by rate of ICA hospitalizations** ^**$**^ **, n (per 100000 IHIO-covered population)**	
Yazd	1376.34
Tehran	647.07
Mazandaran	538.59
Golestan	510.77
Isfahan	482.80
**Hospital ownership, n (%)**	
Governmental academic	106557 (67.19)
Private	34789 (21.94)
Charity	13629 (8.59)
Non-governmental public	2051 (1.29)
Governmental non-academic	1558 (0.98)
**Heart center, n (%)**	
No	117983 (74.40)
Yes	40601 (25.60)
**Hospital bed capacity, n (%)**	
< 100 beds	3790 (2.39)
100–199 beds	47395 (29.89)
200–299 beds	47191 (29.76)
300–399 beds	15817 (9.97)
400–499 beds	15182 (9.57)
>= 500 beds	29209 (18.42)
**Geographic location, n (%)**	
Province center	133183 (83.98)
Other counties	25401 (16.02)
***Clinical and admission***	
**Diagnosis classification (ICD-10), n (%)**	
SIHD	90031 (56.77)
ACS	27008 (17.03)
Unstable angina	7611 (4.80)
NSTEMI	2210 (1.39)
STEMI	4982 (3.14)
MI (unspecified)	4744 (2.99)
Other acute IHD	7461 (4.70)
Other cardiac / vascular	24445 (15.41)
Non-cardiac / other	15013 (9.47)
N/A	2087 (1.32)
**Physician specialty group, n (%)**	
Clinical cardiologist	79784 (50.31)
Interventional cardiologist	58249 (36.73)
Cardiac surgeon	9992 (6.30)
Electrophysiologist	2554 (1.61)
Other / non-cardiac	6380 (4.02)
N/A	1625 (1.02)
**Physician rank, n (%)**	
Fellow	85753 (54.07)
Specialist	66616 (42.01)
Trainee fellow	1978 (1.25)
Trainee specialist	980 (0.62)
GP /other	1632 (1.03)
N/A	1625 (1.02)
**Discharge state, n (%)**	
Full recovery	77849 (49.09)
Partial recovery	69961 (44.12)
Discharge against medical advice	4897 (3.09)
Follow-up	2334 (1.47)
Death	1690 (1.07)
Transfer to another center	1626 (1.03)
Miscellaneous	227 (0.15)
Length of stay, days	1.74 (1.02–3.54)
**Same day discharge, n (%)**	
No	120750 (76.14)
Yes	37834 (23.86)
**Weekend admission, n (%)**	
No	131044 (82.63)
Yes	27540 (17.37)
**Repeat ICA** ^**$#**^ **, n (%)**	
0	140456 (94.09)
1	8376 (5.61)
>= 2	448 (0.29)
Repeat ICA interval^@^, days	27 (12–65)
**28-day repeat ICA** ^**#**^ **, n (%)**	
No	144539 (96.82)
Yes	4741 (3.18)

Values are median (IQR) for continuous variables and n (%) for categorical variables, unless otherwise specified.

Percentages may not sum to 100% due to rounding.

* The other social groups Insurance Fund includes 1,026 foreign nationals.

$ Frequency of all categories available in supplementary materials.

# repeat ICA is defined for patients, not hospitalizations, during the study period (N = 149280).

@ only among patients with repeat ICA (8824).

**Abbreviations:** ICA, Invasive Coronary Angiography; IHIO, Iranian Health Insurance Organization; ICD-10, International Classification of Diseases, 10th Revision; SIHD, Stable Ischemic Heart Disease; ACS, Acute Coronary Syndrome; NSTEMI, Non-ST-Elevation Myocardial Infarction; STEMI, ST-Elevation Myocardial Infarction; MI, Myocardial Infarction; IHD, Ischemic Heart Disease

### 3.2. Revascularization patterns

Revascularization was performed in 74,839 hospitalizations (47.2%). The majority of these procedures were conducted during the index hospitalization (79.2%). PCI was the dominant modality (82.7%). 45.2% of the revascularizations were complex (Table 2). Specific procedural metrics and associated unit costs are summarized in Table 3.

**Table 2:** Procedural characteristics, timing, and complexity of revascularization.

Characteristics	Total (N = 74839)
**Timing, n (%)**	
Index hospitalization	59241 (79.16)
Staged hospitalization	15598 (20.84)
**Type, n (%)**	
PCI	61909 (82.72)
CABG	12587 (16.82)
PCI + CABG	343 (0.46)
**Complex** ^*^ **, n (%)**	
No	41040 (54.84)
Yes	33799 (45.16)
**28-day repeat revascularization** ^**#**^ **, n (%)**	
No	64921 (94.86)
Yes	3515 (5.14)

Values are median (IQR) for continuous variables and n (%) for categorical variables, unless otherwise specified.

Percentages may not sum to 100% due to rounding.

* **Complex Revascularization:** Includes high-intensity surgical and interventional procedures such as extensive bypass grafting (4 + grafts), unprotected left main disease interventions, emergency primary PCI for acute myocardial infarction, and technically demanding procedures like CTO recanalization or multi-vessel/multi-stent single-vessel interventions.

# repeat revascularization is defined for patients, not hospitalizations, during the study period (N = 68436).

**Abbreviations:** PCI, Percutaneous Coronary Intervention; CABG, Coronary Artery Bypass Graft.

**Table 3:** Utilization and unit costs of specific interventional, surgical, and imaging services.

Procedure	Total (N = 158584)	Cost per service, PPP$
**PCI, n (%)**		
Simple single-vessel^*^	34567 (21.80)	–
Balloon	3255 (2.05)	176.8 (137.5–360.7)
1^st^ Stent	31312 (19.75)	356.5 (276.9–713.2)
Complex single-vessel^*^	10314 (6.50)	–
2^nd^ stent	8609 (5.43)	85.7 (64.6–180.3)
3^rd^ stent	1705 (1.07)	57.2 (42.8–121.6)
Multi-vessel^*^	11565 (7.29)	–
Balloon	2453 (1.55)	109.0 (109.0–173.6)
Stent	9112 (5.74)	174.7 (152.7–392.2)
Primary PCI	7706 (4.86)	647.7 (406.3–1148.4)
Unprotected left main PCI	650 (0.41)	361.2 (342.2–601.5)
CTO PCI	1191 (0.75)	381.5 (323.1–901.3)
**CABG, n (%)**		
<= 3 grafts	7250 (4.57)	1477.5 (887.1–2371.9)
> 3 grafts	5680 (3.58)	1302.1 (900.3 - 2552.6)
**Imaging, n (%)**		
Native vessel ICA	153068 (96.52)	149.1 (149.1–412.0)
Bypass vessel ICA	5516 (3.48)	260.2 (260.2–712.3)
Intravascular imaging (OCT/IVUS)	269 (0.17)	92.9 (92.9–174.0)

Values are median (IQR) for continuous variables and n (%) for categorical variables, unless otherwise specified.

Percentages may not sum to 100% due to rounding.

*The cost data is only for the last ballooning or stenting, not total procedure (e.g., multi-vessel).

**Abbreviations:** PCI, Percutaneous Coronary Intervention; CABG, Coronary Artery Bypass Graft; CTO, Chronic Total Occlusion; OCT, Optical Coherence Tomography; IVUS, Intravascular Ultrasound; PPP, Purchasing Power Parity; ICA, Invasive Coronary Angiography.

### 3.3. Inpatient expenditure

The median total cost per inpatient episode was PPP$2,033 (Table 4). Surgical and interventional services (37.1%), along with medications and consumables (35.3%), were the primary drivers of costs. Median out-of-pocket (OOP) payment was PPP$144.1. Aggregated hospitalizations costs amounted to PPP$508,131,974. The primary insurer’s share was PPP$243,805,046 (48.0%), while supplemental health insurance (SHI), institutional deductions and subsidies accounted for PPP$180,096,922 (35.4%). OOP expenditure was PPP$84,230,006 (16.6%). Medications and consumables represented the largest share of total expenditure (36.8%) (S3 Fig in S1 File).

**Table 4:** Economic burden of ICA hospitalizations: total costs, payment distributions, and service-group expenditures.

Cost per hospitalization, PPP$	(N = 158584)
**Total cost, PPP$**	2033.2 (1065.3–4254.5)
**Total cost SIHD, PPP$**	1936.40 (1046.68–4352.35)
**Total cost ACS, PPP$**	2342.21 (1266.88–3967.06)
STEMI	3475.05 (2595.26–4558.85)
NSTEMI	2985.26 (1722.23–4163.44)
Unstable angina	1378.44 (843.82–2670.86)
**Total cost other cardiac, PPP$**	1906.93 (968.75–4204.98)
**Total cost non-cardiac, PPP$**	2188.22 (984.86–4334.61)
**Insurer payment, PPP$**	1005.0 (439.6–1874.9)
**Patient OOP, PPP$**	144.1 (53.3–443.0)
**SHI payment and deductions, PPP$**	623.4 (183.0–1266.7)
**Share of total cost (% of total cost)**	
Insurer share	58.48 (26.73–69.34)
Patient OOP share	7.76 (3.06–16.19)
SHI, deduction, and subsidies share	27.30 (15.70–48.14)
**Total cost per one day of hospitalization, PPP$**	1124.3 (579.7–2314.8)
**Service group cost** ^*^ **, PPP$**	
Accommodation & nursing	345.8 (164.8–632.5)
Diagnostics & imaging	48.1 (19.2–107.7)
Surgical & interventional	741.2 (428.5–1558.2)
Medications & consumables	625.5 (302.2–1801.8)
Professional fees & consultations	3.1 (0.0–51.8)
Specialized & support services	0.6 (0.0–21.2)
**Share of total cost (% of total cost)**	
Accommodation & nursing	17.84 (11.38–26.47)
Diagnostics & imaging	2.43 (0.95–5.13)
Surgical & interventional	37.14 (26.59–47.85)
Medications & consumables	35.34 (25.20–47.73)
Professional fees & consultations	0.19 (0.00–1.84)
Specialized & support services	0.04 (0.00–0.67)

Values are median (IQR) for continuous variables and n (%) for categorical variables, unless otherwise specified.

Percentages may not sum to 100% due to rounding.

* Each group contains specific services: **1) Accommodation & nursing:** Hoteling, Nursing care packages and services, and Companion cost; **2) Diagnostics & imaging**: Routine lab tests, Genetic tests, Pathology, X-ray, Ultrasound, CT scan, MRI, Nuclear medicine, Ancillary diagnostic procedures, Bone Mineral Densitometry (BMD), Eye diagnostics services; **3) Surgical & interventional:** Pulmonary interventions, Vascular interventions, Surgery services, Angiography, Digital angiography; **4) Medications & consumables:** Ward medications and consumables, Operating room medications and consumables, and Prosthesis/orthosis; **5) Professional fees & consultations:** Visit fees, Internal medicine and Consultation services; **6) Specialized & support services:** Optometry, Audiology, Rehabilitation, Physiotherapy, Occupational therapy, Speech therapy, Radiotherapy, Chemotherapy, Dentistry services, Dialysis, Forensic medicine, Ambulance, Blood transfer, and Other services.

**Abbreviations:** ACS, acute coronary syndrome; MI, myocardial infarction; NSTEMI, non-ST-elevation MI; OOP, Out of Pocket; SIHD, stable ischemic heart disease; STEMI: ST elevation MI; SHI, Supplemental Health Insurance; PPP, Purchasing Power Parity.

### 3.4. Determinants of ICA volume and rate

Uni- and multivariate negative binomial regression identified critical hospital-level predictors of angiography volume (S4 and S5 Figs, S3 Table in S1 File). Heart centers experienced a 2.2-fold higher volume (IRR: 2.2; 95% CI: 1.4–3.7; p = 0.001). Private (IRR: 0.59; p = 0.006) and Non-Governmental Public (IRR: 0.21; p < 0.001) ownership were associated with lower volumes compared to academic centers (S4 and S5 Figs in S1 File). At the provincial level (S6 Fig, S4 Table in S1 File), ICA rates were positively associated with healthcare infrastructure: density of angiography devices (IRR: 1.3; 95% CI: 1.1–1.5; p = 0.01) and active beds (IRR: 2.3; 95% CI: 1.1–4.8; p = 0.03). A higher provincial deprivation rank (less deprivation) was associated with increased ICA rates (IRR: 1.1; 95% CI: 1.0–1.1; p < 0.001). These factors contributed to marked regional disparities (S5 Table in S1 File).

### 3.5. Multi-pollutant mixture analysis

WQS regression identified a significant positive association between the air pollution mixture and ICA rates among ACS patients (IRR: 1.10; 95% CI: 1.07–1.14 per quartile increase in exposure). Carbon monoxide (CO; 49.5%), sulfur dioxide (SO2; 30.1%), and PM2.5 (20.5%) were the major contributors (S7 Fig in S1 File).

### 3.6. Determinants of revascularization and LOS

**Fig 1:**
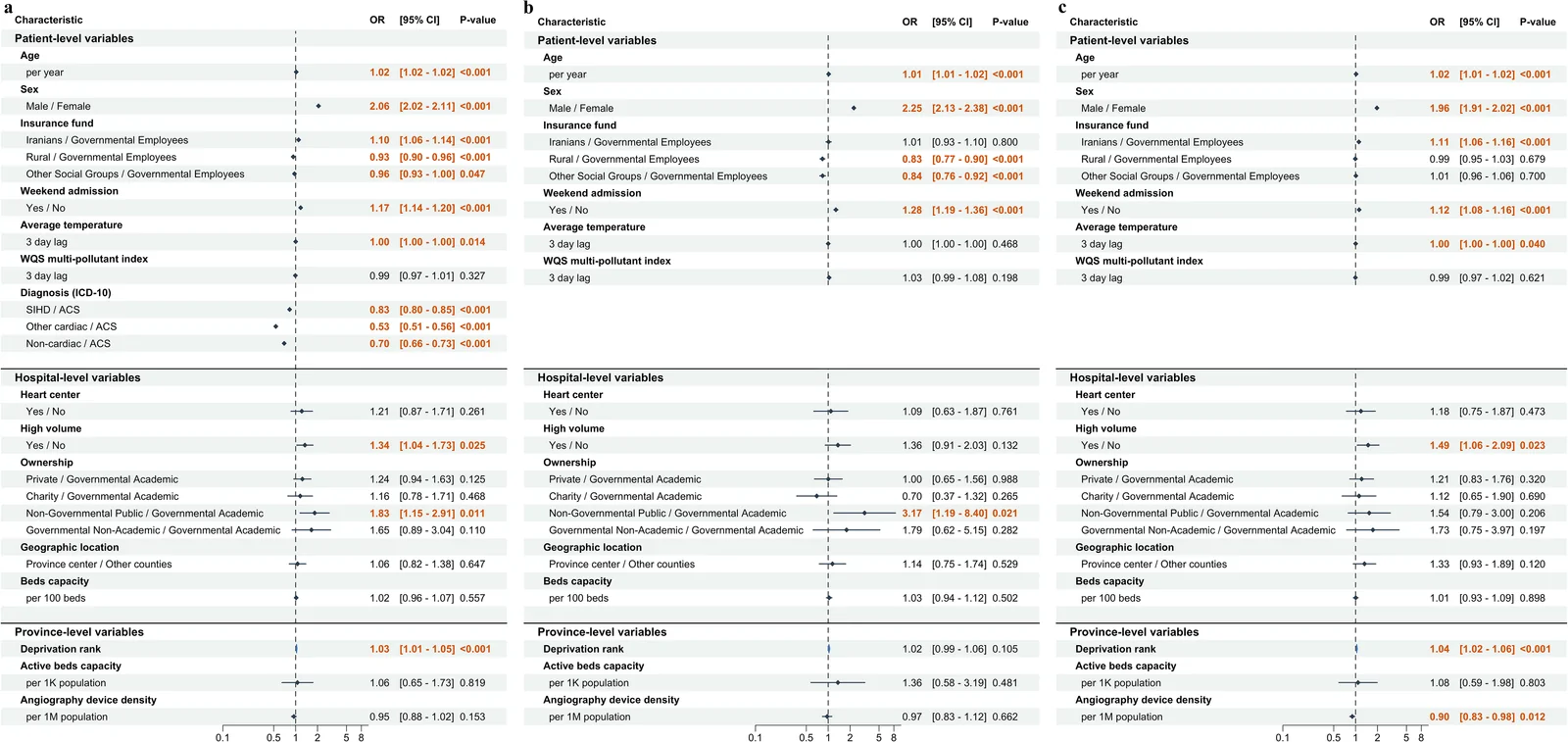
Hierarchical mixed-effects models for determinants of revascularization across clinical subgroups (a:total, b:ACS, c:SIHD). **Abbreviations:** ACS, Acute Coronary Syndrome; SIHD, Stable Ischemic Heart Disease; WQS, Weighted Quantile Sum.

**Fig 2:**
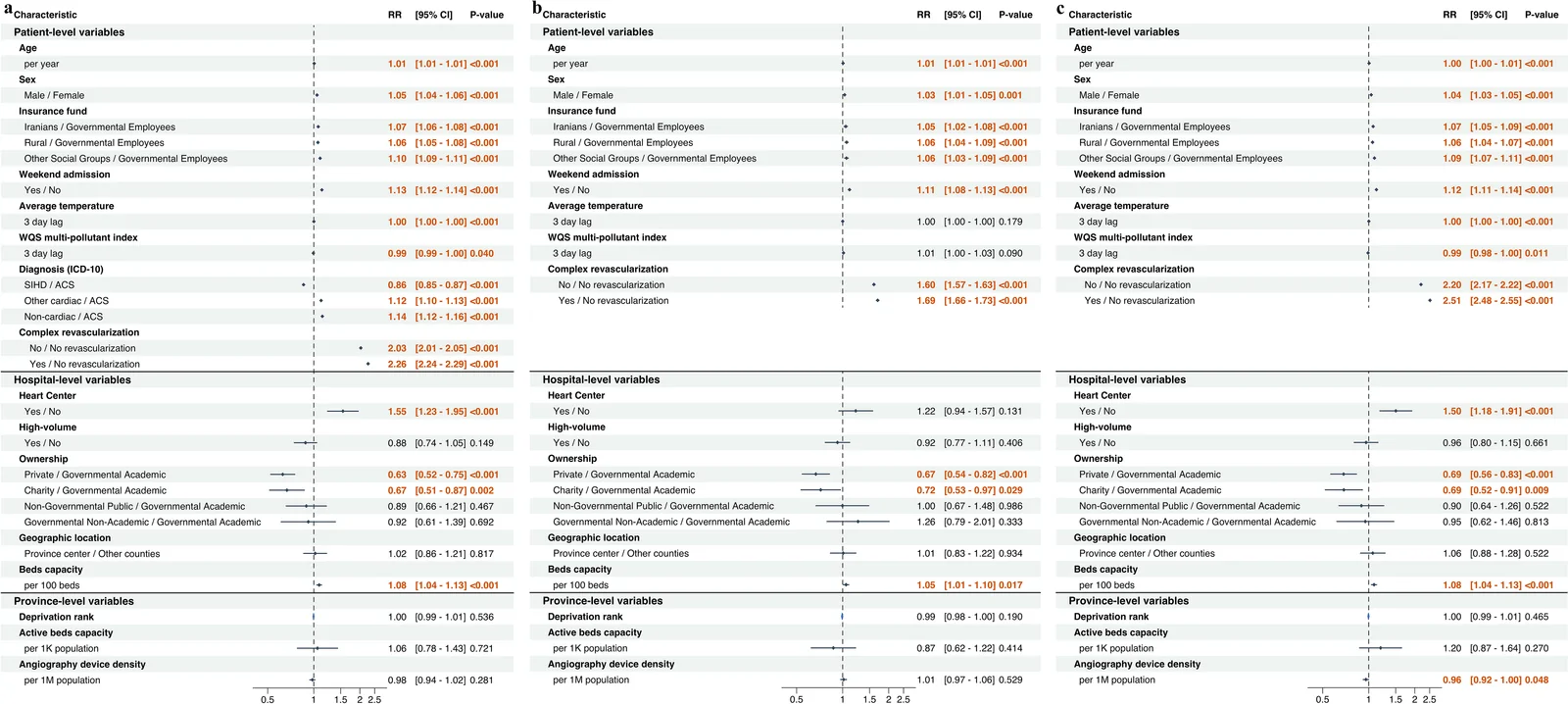
Hierarchical mixed-effects models for determinants of length of stay (LOS) across clinical subgroups (a:total, b:ACS, c:SIHD). **Abbreviations:** ACS, Acute Coronary Syndrome; SIHD, Stable Ischemic Heart Disease; WQS, Weighted Quantile Sum.

- **Patient-level factors:** Male gender and older age were associated with increased revascularization and LOS (Figs 1 and 2). Weekend admissions were associated with higher odds of revascularization for ACS (OR: 1.28; 95% CI: 1.19–1.36) and extended total LOS (1.13; 95% CI 1.12–1.14). Patients with SIHD (OR: 0.83; 95% CI: 0.80–0.85) or non-cardiac diagnoses (OR: 0.70; 95% CI: 0.66–0.73) were less likely to undergo revascularization than those with ACS. LOS was shorter for SIHD (RR: 0.86; 95% CI 0.85–0.87), but extended for non-cardiac hospitalizations (RR: 1.14; 95% CI 1.12–1.16). Revascularizations – particularly those deemed complex – were associated with extended LOS (RR: 2.26; 95% CI 2.24–2.29). (all p-values < 0.001)

- **Systemic disparities:** Systemic determinants for revascularization varied sharply by subgroup. Deprivation rank (OR: 1.04; 95% CI: 1.02–1.06; p < 0.001) and high-volume status (OR: 1.49; 95% CI: 1.06–2.09; p = 0.02) were predictors of revascularization for SIHD, yet neither reached significance in the ACS group (p = 0.11 and p = 0.76, respectively). Angiography device density was negatively associated with revascularization for only SIHD patients (OR: 0.90; 95% CI: 0.83–0.98; p = 0.01). Total LOS was markedly longer for heart centers (RR: 1.55; 95% CI 1.23–1.95), and shorter in private (RR: 0.63; 95% CI 0.52–0.75) and charity (RR: 0.67; 95% CI 0.51–0.87) hospitals (p-values < 0.001).

### 3.7. Determinants of hospitalization costs

**Fig 3:**
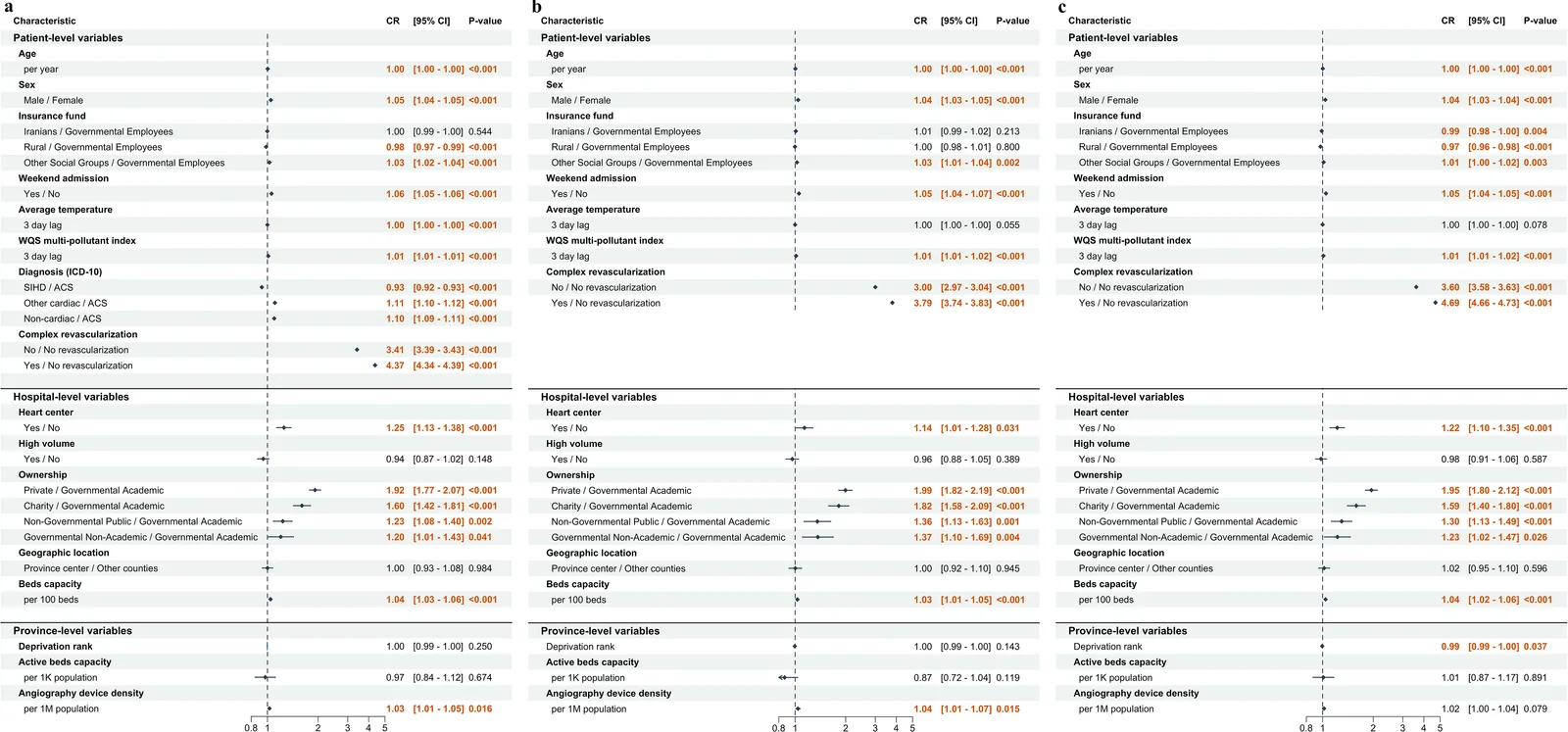
Hierarchical mixed-effects models for determinants of total hospitalization costs across clinical subgroups (a:total, b:ACS, c:SIHD). **Abbreviations:** ACS, Acute Coronary Syndrome; SIHD, Stable Ischemic Heart Disease; WQS, Weighted Quantile Sum.

Costs were highly sensitive to revascularization complexity and facility characteristics. Complex revascularization was the primary driver of cost. Complexity increased total costs by a factor of 4.37 (95% CI: 4.34–4.39). Private hospital ownership nearly doubled costs across all groups (total cohort CR: 1.92; 95% CI: 1.77–2.07). Bed capacity consistently predicted higher costs (total cohort CR: 1.04; 95% CI: 1.03–1.06). This was also observed for heart centers (total cohort CR: 1.25; 95% CI 1.13–1.38). Male gender and higher age were also associated with higher costs (all p-values < 0.001) (Fig 3).

### 3.8. Model performance

Hierarchical models demonstrated high explanatory power, with the conditional R^2^ reaching up to 0.24, 0.47, and 0.79 for revascularization, LOS, and cost models, respectively. Inclusion of systemic factors enhanced predictive capability in the revascularization, LOS, and cost models, where marginal R^2^ increased by up to 2.9, 13.6, and 11.9 percentage points. ICC analysis highlighted the dominant role of the hospital level, which accounted for 8.2% (95% CI 5.8%−9.2%), 19.5% (95% CI 14.5%−21.3%), and 11.0% (95% CI 7.9%−12.6%) of the variation in revascularization rate, LOS, and cost respectively. Model diagnostics via DHARMa residual simulations and VIF tests confirmed model stability across all clinical subgroups (S6 Table in S1 File).

## 4. Discussion

Aggregate ICA hospitalization costs were in excess of PPP$508 million during the study period, with a median of PPP$2033 per hospitalization. These costs are far below estimates for comparable services in developed countries [6,7,34]. This may, in part, be attributed to subsidies for care provided to governmental facilities – covering up to a third of inpatient costs [15]. A relatively low uptake of more advanced (and costly) radiological and procedural services – due to lower availability or differences in practice patterns – may have also contributed to lower estimates for upfront costs [35]. The OOP share (17%) was also lower than reported in previous studies – indicating a relatively higher coverage for the costs of coronary care. The most prominent portions of costs were procedural (37%) and medical (35% drug and consumables). A small portion (< 1%) of costs was attributable to rehabilitative services. This reiterates the low national uptake for these services [15,36]. We also observed an extremely low (< 0.2%) uptake for intracoronary imaging. This is consistent with disparities in access to such services – associated with improved clinical outcomes [7,35].

SIHD was the most frequent indication for ICA. The ACS/SIHD ratio for ICAs in our cohort was lower than reported in studies from developed nations [6,37]. This may suggest suboptimal patient selection for ICA. Less widespread utilization of non-invasive coronary imaging in SIHD may have also contributed to these observations [38]. With this, the rate of revascularization was comparable [39] – suggesting a possible accompanying overutilization of revascularization services. Revascularization odds were highest in the ACS subgroup, while patients undergoing ICA during non-cardiac hospitalizations incurred higher costs. This may have followed the presence of acute and chronic comorbidities – as indicated by extended LOS – in these cases.

Significant temporal patterns were noted in ICA hospitalizations. As expected, a higher ACS/SIHD ratio for ICAs was noted on weekends and during the end of year period. Revascularization odds were also higher in weekend hospitalizations. This is consistent with previously described characteristics for weekend hospitalizations [40,41]. Although the frequently discussed “weekend effect” on patient outcomes has been found to be minimal [42], our findings suggest persistent negative economic effects. In addition to the difference in clinical profile (selection of emergent cases), prolonged stays due to a lower likelihood of weekend discharge were associated with these observations [40,41].

The WQS analysis of ICA rates due to ACS suggested significant positive contributions from ambient pollutants. This is consistent with previous literature. Extreme temperatures and ambient air pollution have been consistently linked with higher rates of hospitalization and mortality due to acute IHD [43,44]. Despite a large historic focus on PM and NO_x_ pollution, our analysis suggests a higher contribution from CO and sulfurous pollution – in line with recent findings [45,46]. However, we observed a null or minimal effect for changes in ambient temperature and air pollution on the odds of revascularization. This may be due to a concomitant increases in clinical conditions with overlapping symptomatology (e.g., angina mimics and respiratory difficulty), and/or more pronounced exacerbations of non-obstructive coronary disease [47–49].

Older and male patients comprised a majority of the study cohort. Older age and male gender were also associated with both higher odds of revascularization, prolonged LOS, and overall inpatient costs. These findings are consistent with previous studies. In addition to a higher likelihood of IHD and revascularization requirement [3,11], frailty and comorbidities lead to a higher rate of complications. These contribute to higher LOS and aggregate inpatient costs [50]. Male gender is similarly associated with higher burden of IHD [3,11]. While, more “typical” presentations contribute to the likelihood of early diagnosis of acute coronary episodes in male patients – leading to a higher rate of ICAs with subsequent revascularization [51].

Coverage by different insurance funds was associated with significant variations in service utilization. Vulnerable patients covered by the Other Social Group Fund incurred higher inpatient costs despite specific subsidies – associated with lower costs for general IHD care [13,15] – and had lower revascularization odds. LOS was markedly prolonged in these patients. Patients covered by the Rural Fund also experienced prolonged hospitalizations and lower revascularization odds, with the former being a source of divergence in our findings compared to studies from developed nations [52,53]. These suggest higher rates of comorbidities and complications in the setting of more advanced coronary care following delayed care-seeking or poor access [53,54]. Delayed discharge due to providers’ concerns for future care and accessibility, and/or prolonged recovery following more conservative treatments may have also contributed to prolonged LOS [53,55,56]. Hospital ownership was similarly a predictor of costs. Hospitalizations in non-academic centers were associated with higher costs. For non-governmental public centers (including those operated by Social Security and Military hospitals), this may be attributable to selection of more severe cases [15,57] – as supported by an observation of higher ACS revascularization. Expectedly, hospitalizations in private centers were associated with higher costs and shorter LOS; likely due to preferences for high turnover non-emergent procedural services [58]. Hospitalizations in heart centers (specialty hospitals) were associated with higher costs and markedly extended LOS. Provision of service to more complex cases (higher comorbidities) and higher utilization of novel diagnostic and/or procedural services may explain this finding [59,60]. Revascularization odds, however, were not higher for these centers. This may have followed a larger focus on subsequent stages of care for referral cases [60,61].

On a provincial level, socioeconomic deprivation was associated with lower revascularization odds – particularly among patients with SIHD. This may be due to the migration of patients from less developed provinces for ICA with “semi-elective” or “elective” indications. Lack of access to specialized care or providers’ referral of select patients – with higher procedural risk – may have motivated this phenomenon [62]. Although, our findings regarding costs and LOS are somewhat contradictory to such an appraisal. We did not observe costlier or prolonged hospitalizations (delayed/complex case profile) in less deprived provinces. More likely, mobility for elective SIHD care may have followed patient preference [63,64]. Overutilization of ICA and revascularization services in less deprived provinces, per se, is also a possibility [65]. ICAs in provinces with higher device density (ad hoc marker for medical access) were associated with lower odds of SIHD revascularization and higher inpatient costs. The observation may have followed higher availability and utilization of novel diagnostic (complex case-selection for revascularization) and procedural options leading to higher upfront costs [66].

### 4.1. Limitations and considerations

This study benefits from the inclusion of data from a large-scale national cohort. However, our findings regarding drivers of cost and service utilization should be interpreted according to the profile of the studied cohort. Although optimal for organizational decision-making, indirectness should be considered for policymaking on a national level. In particular, the IHIO covers a high proportion of patients with lower socioeconomic status from backgrounds with limited access to care [17]. The predominance of governmental academic facilities may also skew the summary reports, including a perception of lower overall costs. At the same time, regional and systemic disparities may be less pronounced in our study, owing to a more homogeneous patient profile. Our study focuses on patients undergoing ICA. Therefore, we do not provide direct estimates on the determinants of inpatient costs for IHD care or the utilization of services such as non-invasive coronary imaging. The exploratory models also focus on revascularization and LOS as major contributors to costs – the diagnostic and medical costs of comorbidities and complications being assumed to be correlated with LOS [67]. The specifics of these costs have been left unexplored due to lack of reliable data on indications for service utilization, or complication rates. This, in tandem with lack of reliable information on patient characteristics and long-term outcomes, prevents an assessment of the appropriateness of service utilizations and care-seeking behaviors. It should also be noted that the lack of inclusion of individual patient characteristics (comorbidities, direct indicators of socioeconomic status, ethnicity, etc.) diminishes overall model performance and explanatory power, while allowing for a better illustration of the effects of systemic parameters. Adjustment for these variables would minimize disparities in systemic factors mediated by distinct case-selection. In essence, our study describes systemic disparities assuming a non-modifiable patient profile for different facilities/provinces. Furthermore, treating provincial deprivation as a continuous rank variable assumes proportional spacing between ranks; this approach may mask non-linear threshold effects in regional disparities. Finally, the study design precludes us from rendering definitive judgments on causality. We also recognize the inherent heterogeneity within the complex revascularization category, which encompasses both advanced interventional and surgical procedures. While our hierarchical models identify complexity as the primary driver of cost, this effect size reflects the cumulative resource intensity—including specialized consumables, prolonged ICU occupancy, and professional expertise—shared by these high-stakes procedures. The specific unit costs provided in Table 3 further contextualize these findings. Altogether, our findings serve as a unique and valuable, yet preliminary basis for much needed future research. This includes studies on geographical and socioeconomic disparities, along with microeconomics and cost-effectiveness analyses for particular services.

In conclusion, we evaluated overall inpatient costs, service uptake and drivers of costs, and systemic and demographic determinants associated with higher costs in a nationwide cohort of patients undergoing ICA. Significant systemic disparities were identified in the economic burden of coronary care. The results provide valuable insights as to the pattern of service utilization in a developing nation. These findings are critical for identifying future funding and research priorities. We identified distinct cost profiles for different centers – possibly due to specific case-selection and patient profiles. Our findings also indicate a possible underutilization of non-invasive diagnostic alternatives to ICA, intracoronary imaging, and rehabilitative services. Significant regional disparities – driven by socioeconomic deprivation and medical access – were also identified.

## Supporting information

S1 File Supplementary Figures and Tables. This file contains all supplementary figures and tables supporting the main text.
(DOCX)

S1 Fig Graphical Abstract. (TIF)
