# Supplementary material for: Economic drivers and systemic disparities in invasive coronary angiography: Insights from a national claims database
Source: PLoS One. 2026 Sep 3;21(9):e0356496. doi: 10.1371/journal.pone.0356496 (PMC13541126; doi:10.1371/journal.pone.0356496)
Supplement: Supplementary file 1 [file pone.0356496.s001.docx]

## *Supplementary Materials*

## Economic drivers and systemic disparities in invasive coronary angiography: Insights from a national claims database

**Supplementary Figures**

**Fig S1. Monthly temporal trends in total invasive coronary angiography admissions.**

**Fig S2.** **Frequency distribution of repeated invasive coronary angiography procedures per patient.**

**Fig S3. Contributors to aggregate hospitalization costs in the study cohort.**

**Fig S4.** **Univariate analysis of hospital characteristics associated with invasive coronary angiography rate.**

**Fig S5.** **Multivariate predictors of hospital-level invasive coronary angiography volume.**

**Fig S6.** **Univariate association between provincial socioeconomic infrastructure and invasive coronary angiography rates.**

**Fig S7.** **Relative weights of air pollutants in the weighted quantile sum (WQS) regression index for ACS Patients.**

**Supplementary Tables**

**Table S1.** **ICD-10 coding definitions used for clinical cohort classification.**

| Main diagnosis | Sub-diagnosis | Specific ICD-10 Codes Included |
| --- | --- | --- |
| **Acute Coronary Syndrome (ACS)** | Unstable Angina | I20.0 |
|  | STEMI | I21.0, I21.1, I21.2, I21.3 |
|  | NSTEMI | I21.4 |
|  | MI (Unspecified) | I21, I21.9 |
|  | Subsequent / Recurrent MI | I22, I22.0, I22.1, I22.8, I22.9 |
|  | Other Acute IHD | I24, I24.0, I24.1, I24.8, I24.9 |
| **Stable Ischemic Heart Disease (SIHD)** | Stable Angina | I20, I20.1, I20.8, I20.9 |
|  | Chronic CAD / Atherosclerosis | I25, I25.0, I25.1, I25.3, I25.4, I25.5, I25.6, I25.8, I25.9 |
|  | History of Old MI | I25.2 |
|  | CABG + Chronic CAD | I25.7 |
| **Other Cardiac / Vascular** | Broad category including: Hypertensive, Rheumatic, Pulmonary, Cerebrovascular, and other structural/vascular diseases | I00-I15, I26-I99, Q20-Q28 (excluding primary IHD codes above) |
| **Non-Cardiac / Other** | Broad category including: Non-cardiovascular medical, surgical, and external causes of admission | A00-T98, Z00-Z99 (excluding cardiovascular and vascular subsets) |

**Abbreviations:** ICD, International Classification of Disease; STEMI, ST-Elevation Myocardial Infarction; NSTEMI, Non-ST-Elevation Myocardial Infarction; MI, Myocardial Infarction; IHD, Ischemic Heart Disease; CAD, Coronary Artery Disease; CABG, Coronary Artery Bypass Graft.

**Table S2.** **Temporal trends in the ratio of elective (SIHD) to urgent/emergent (ACS) angiography by weekday and month.**

| Weekday | SIHD / ACS ratio | P-value | Pairwise difference |
| --- | --- | --- | --- |
| Monday | 3.63 | **< 0.01** | Friday, Saturday, Wednesday |
| Tuesday | 3.62 |  | Friday, Saturday, Wednesday |
| Wednesday | 3.25 |  | Friday, Monday, Tuesday |
| Thursday | 3.44 |  | Friday |
| Friday | **2.12** |  | With all other days |
| Saturday | 3.30 |  | Friday, Monday, Tuesday |
| Sunday | 3.44 |  | Friday |
| Month | **SIHD / ACS ratio** | **P-value** | **Pairwise difference** |
| January | 3.40 | **< 0.01** | March |
| February | 3.13 |  | March, April, July, May |
| March | **2.68** |  | With all other months |
| April | 3.66 |  | March, February, September |
| May | 3.67 |  | March, February, September, October |
| June | 3.32 |  | March |
| July | 3.54 |  | March, February |
| August | 3.41 |  | March |
| September | 3.15 |  | March, April, May |
| October | 3.26 |  | March, May |
| November | 3.50 |  | March |
| December | 3.26 |  | March, May |

**Table S3. Hospital-level factors associated with angiography volume: univariate and multivariate analyses.**

| Characteristic | Total (N = 145) | Univariable IRR (95% CI) | P-value | Multivariable IRR (95% CI) | P-value |
| --- | --- | --- | --- | --- | --- |
| Ownership, n (%) | | | | | |
| Governmental Academic | 73 (50.34) | 1.00 (Reference) | - | 1.00 (Reference) | - |
| Non-Governmental Public | 8 (5.52) | **0.18 (0.10 – 0.36)** | **<0.01** | **0.21 (0.11 – 0.42)** | **<0.01** |
| Governmental Non-Academic | 4 (2.76) | **0.27 (0.12 – 0.76)** | **<0.01** | **0.35 (0.16 – 0.94)** | **0.02** |
| Private | 49 (33.79) | **0.49 (0.35 – 0.68)** | **<0.01** | **0.59 (0.41 – 0.85)** | **0.01** |
| Charity | 11 (7.59) | 0.85 (0.50 – 1.57) | 0.57 | 0.92 (0.54 – 1.70) | 0.79 |
| Heart center, n (%) | | | | | |
| No | 129 (88.97) | 1.00 (Reference) | - | 1.00 (Reference) | - |
| Yes | 16 (11.03) | **2.77 (1.76 – 4.64)** | **<0.01** | **2.22 (1.39 – 3.69)** | **<0.01** |
| Geographic location, n (%) | | | | | |
| Other counties | 26 (17.93) | 1.00 (Reference) | - | 1.00 (Reference) | - |
| Province center | 119 (82.07) | 1.31 (0.87 – 1.92) | 0.18 | 1.26 (0.86 – 1.81) | 0.23 |
| Beds Capacity, Median (IQR) | 222 (156 – 370) | 1.05 (0.98 – 1.15)^*^ | 0.17 | 1.02 (0.94 – 1.11)^*^ | 0.64 |
| Beds Capacity, n (%) | | | | | |
| <100 | 6 (4.14) | - | - | - | - |
| 100-199 | 51 (35.17) | - | - | - | - |
| 200-299 | 42 (28.97) | - | - | - | - |
| 300-399 | 11 (7.59) | - | - | - | - |
| 400-499 | 13 (8.97) | - | - | - | - |
| >= 500 | 22 (15.16) | - | - | - | - |
| ICA volume, Median (IQR) | 878 (416-1358) | - | - | - | - |

*per 100 beds

Values are median (IQR) for continuous variables and n (%) for categorical variables, unless otherwise specified

**Abbreviations:** IRR, Incidence Rate Ratio; CI, Confidence Interval; IQR, Interquartile Range

**Model Selection:** For count, due to significant overdispersion of the dependent variable (angiography count), Negative Binomial regression was utilized instead of Poisson regression.

**Multivariable model performance:** AIC:2290, R2 Nagelkerke:0.41

**Table S4.** **Provincial socioeconomic and healthcare infrastructure predictors of angiography rates.**

| Characteristic | Total (N = 31) | Univariate IRR (95% CI) | P-value | McFadden R^2^ |
| --- | --- | --- | --- | --- |
| Socioeconomic Factors | | | | |
| Deprivation rank | - | **1.06 (1.04 – 1.09)** | **<0.01** | **0.05** |
| Active beds per 1000 population (Mean ± SD) | 1.75 ± 0.30 | **2.31 (1.11 – 4.77)** | **0.03** | **0.01** |
| Angiography device per one million population | 2.60 (2.00 - 3.10) | **1.25 (1.06 - 1.48)** | **0.01** | **0.02** |
| ICA rate (per 100000 IHIO covered population) | 249.39 (200.15 - 435.28) | - | - | - |

Values are median (IQR) for continuous variables and n (%) for categorical variables, unless otherwise specified

**Abbreviations:** IRR, Incidence Rate Ratio; CI, Confidence Interval; IQR, Interquartile Range

**Model Selection:** Due to continuous non-negative and skewed distribution of ICA rate and to provide a parsimonious and excellent fit for population-standardized data a GLM with Gamma distribution and a Log link was preferred.

**Table S5.** **Geographical distribution and provincial rates of invasive coronary angiography (ICA) and revascularization.**

| Province | ICA per 100000 IHIO-covered population (n) | Total ICA, n (%) | Revascularization per 100000 IHIO-covered population (n) | Total Revascularization, n (%) | Revascularization rate (%) |
| --- | --- | --- | --- | --- | --- |
| Yazd | 1376.3 | 4419 (2.79) | 699.9 | 2247 (3.00) | 50.85 |
| Tehran | 647.1 | 32181 (20.29) | 298.4 | 14839 (19.83) | 46.11 |
| Mazandaran | 538.6 | 9202 (5.80) | 210.8 | 3602 (4.81) | 39.14 |
| Golestan | 510.8 | 6454 (4.07) | 202.3 | 2556 (3.42) | 39.60 |
| Isfahan | 482.8 | 9212 (5.81) | 268.7 | 5126 (6.85) | 55.64 |
| Guilan | 469.0 | 7024 (4.43) | 239.3 | 3584 (4.79) | 51.03 |
| Fars | 462.2 | 14229 (8.97) | 217.7 | 6703 (8.96) | 47.11 |
| Alborz | 442.6 | 4000 (2.52) | 209.7 | 1895 (2.53) | 47.38 |
| Razavi Khorasan | 428.0 | 19096 (12.04) | 218.9 | 9768 (13.05) | 51.15 |
| Qom | 377.3 | 2398 (1.51) | 139.2 | 885 (1.18) | 36.91 |
| Semnan | 374.2 | 946 (0.60) | 135.7 | 343 (0.46) | 36.26 |
| Lorestan | 338.9 | 4373 (2.76) | 145.6 | 1879 (2.51) | 42.97 |
| East Azerbaijan | 319.3 | 7556 (4.76) | 193.5 | 4579 (6.12) | 60.60 |
| Ardabil | 273.2 | 2417 (1.52) | 126.4 | 1118 (1.49) | 46.26 |
| Kermanshah | 271.0 | 3670 (2.31) | 134.4 | 1820 (2.43) | 49.59 |
| Kerman | 249.4 | 4429 (2.79) | 110.7 | 1966 (2.63) | 44.39 |
| Zanjan | 246.6 | 1629 (1.03) | 125.5 | 829 (1.11) | 50.89 |
| Khuzestan | 232.3 | 6589 (4.15) | 81.0 | 2297 (3.07) | 34.86 |
| Qazvin | 227.2 | 1397 (0.88) | 99.2 | 610 (0.82) | 43.66 |
| South Khorasan | 225.8 | 1176 (0.74) | 95.3 | 496 (0.66) | 42.18 |
| Bushehr | 219.4 | 1064 (0.67) | 102.7 | 498 (0.67) | 46.80 |
| Hamedan | 214.9 | 2623 (1.65) | 120.0 | 1465 (1.96) | 55.85 |
| Chaharmahal and Bakhtiari | 206.2 | 1350 (0.85) | 119.6 | 783 (1.05) | 58.00 |
| West Azerbaijan | 194.1 | 4928 (3.11) | 85.5 | 2172 (2.90) | 44.07 |
| Markazi | 181.8 | 1173 (0.74) | 110.5 | 713 (0.95) | 60.78 |
| Hormozgan | 140.9 | 1639 (1.03) | 68.1 | 792 (1.06) | 48.32 |
| North Khorasan | 120.8 | 884 (0.56) | 46.9 | 343 (0.46) | 38.80 |
| Ilam | 115.9 | 416 (0.26) | 13.6 | 49 (0.07) | 11.78 |
| Kurdistan | 65.7 | 770 (0.49) | 35.0 | 411 (0.55) | 53.38 |
| Sistan and Baluchestan | 45.1 | 1228 (0.77) | 17.3 | 470 (0.63) | 38.27 |
| Kohgiluyeh and Boyer-Ahmad | 21.6 | 112 (0.07) | 0.2 | 1 (0.00) | 0.89 |
| Iran | 348.4 | 158584 (100) | 164.4 | 74839 (100) | 47.19 |

**Table S6.** **Collinearity diagnostics (Variance Inflation Factors) for the hierarchical mixed-effects models (Total Cohort).**

| Variable | Revascularization (VIF) | Length of Stay (VIF) | Total Cost (VIF) |
| --- | --- | --- | --- |
| Patient-Level | | | |
| Age | 1.06 | 1.06 | 1.06 |
| Sex | 1.03 | 1.06 | 1.06 |
| Insurance fund | 1.07 | 1.07 | 1.07 |
| Weekend admission | 1.00 | 1.00 | 1.00 |
| Average temperature | 1.04 | 1.04 | 1.04 |
| WQS multi-pollutant index | 1.04 | 1.04 | 1.04 |
| Diagnosis (ICD-10) | 1.00 | 1.01 | 1.02 |
| Complex revascularization | NA | 1.05 | 1.05 |
| Hospital-Level | | | |
| Heart center | 1.37 | 1.36 | 1.42 |
| High-volume | 1.44 | 1.42 | 1.46 |
| Ownership | 1.98 | 2.05 | 1.97 |
| Geographic location | 1.21 | 1.22 | 1.22 |
| Beds capacity | 1.53 | 1.57 | 1.50 |
| Province-Level | | | |
| Deprivation Rank | 1.43 | 1.62 | 1.51 |
| Active beds capacity | 1.52 | 2.00 | 1.58 |
| Angiography device density | 1.88 | 2.56 | 2.04 |

**Abbreviations:** VIF, Variance Inflation Factor; WQS, Weighted Quantile Sum; ICD, International Classification of Disease.
