# Supplementary material for: Economic drivers and systemic disparities in invasive coronary angiography: Insights from a national claims database
Source: PLoS One. 2026 Sep 3;21(9):e0356496. doi: 10.1371/journal.pone.0356496 (PMC13541126; doi:10.1371/journal.pone.0356496)
Supplement: Peer review history

# Peer review: Decision Letter 0

**DOI:** 10.1371/journal.pone.0356496.r001

**Authors:** Hossein Ali Adineh

17 Jun 2026

Dear Dr. Semnani,

Thank you for submitting your manuscript to PLOS ONE. After careful consideration, we feel that it has merit but does not fully meet PLOS ONE’s publication criteria as it currently stands. Therefore, we invite you to submit a revised version of the manuscript that addresses the points raised during the review process.

Please submit your revised manuscript by Aug 01 2026 11:59PM. If you will need more time than this to complete your revisions, please reply to this message or contact the journal office at plosone@plos.org. When you're ready to submit your revision, log on to https://www.editorialmanager.com/pone/ and select the 'Submissions Needing Revision' folder to locate your manuscript file.

- A letter that responds to each point raised by the academic editor and reviewer(s). You should upload this letter as a separate file labeled 'Response to Reviewers'.
- A marked-up copy of your manuscript that highlights changes made to the original version. You should upload this as a separate file labeled 'Revised Manuscript with Track Changes'.
- An unmarked version of your revised paper without tracked changes. You should upload this as a separate file labeled 'Manuscript'.

If you would like to make changes to your financial disclosure, please include your updated statement in your cover letter. Guidelines for resubmitting your figure files are available below the reviewer comments at the end of this letter.

If applicable, we recommend that you deposit your laboratory protocols in protocols.io to enhance the reproducibility of your results. Protocols.io assigns your protocol its own identifier (DOI) so that it can be cited independently in the future. For instructions see: https://journals.plos.org/plosone/s/submission-guidelines#loc-laboratory-protocols. Additionally, PLOS ONE offers an option for publishing peer-reviewed Lab Protocol articles, which describe protocols hosted on protocols.io. Read more information on sharing protocols at https://plos.org/protocols?utm_medium=editorial-email&utm_source=authorletters&utm_campaign=protocols.

As the corresponding author, your ORCID iD is verified in the submission system and will appear in the published article. PLOS supports the use of ORCID, and we encourage all coauthors to register for an ORCID iD and use it as well. Please encourage your coauthors to verify their ORCID iD within the submission system before final acceptance, as unverified ORCID iDs will not appear in the published article. *Only*  the individual author can complete the verification step; PLOS staff *cannot*  verify ORCID iDs on behalf of authors.

We look forward to receiving your revised manuscript.

Kind regards,

Hossein Ali Adineh, Ph.D

Academic Editor

PLOS One

Journal Requirements:

When submitting your revision, we need you to address these additional requirements.

1. Please ensure that your manuscript meets PLOS ONE's style requirements, including those for file naming. The PLOS ONE style templates can be found at

https://journals.plos.org/plosone/s/file?id=wjVg/PLOSOne_formatting_sample_main_body.pdf and https://journals.plos.org/plosone/s/file?id=ba62/PLOSOne_formatting_sample_title_authors_affiliations.pdf

2. Your ethics statement should only appear in the Methods section of your manuscript. If your ethics statement is written in any section besides the Methods, please delete it from any other section.

3. In the online submission form, you indicated that the data that support the findings of this study are held by the Iran Health Insurance Organization (IHIO) and are subject to legal and national security restrictions. The authors were granted access under a specific research agreement and are not legally permitted to share the data publicly or with third parties. Data access requests can be directed to the Research Committee of the Iran Health Insurance Organization (https://nchir-r.ihio.gov.ir/general/homePage.action, Email: intl@ihio.gov.ir).

All PLOS journals now require all data underlying the findings described in their manuscript to be freely available to other researchers, either 1. In a public repository, 2. Within the manuscript itself, or 3. Uploaded as supplementary information.

This policy applies to all data except where public deposition would breach compliance with the protocol approved by your research ethics board. If your data cannot be made publicly available for ethical or legal reasons (e.g., public availability would compromise patient privacy), please explain your reasons on resubmission and your exemption request will be escalated for approval.

4. Please include captions for your Supporting Information files at the end of your manuscript, and update any in-text citations to match accordingly. Please see our Supporting Information guidelines for more information: http://journals.plos.org/plosone/s/supporting-information.

5. If the reviewer comments include a recommendation to cite specific previously published works, please review and evaluate these publications to determine whether they are relevant and should be cited. There is no requirement to cite these works unless the editor has indicated otherwise.

6. Please review your reference list to ensure that it is complete and correct. If you have cited papers that have been retracted, please include the rationale for doing so in the manuscript text, or remove these references and replace them with relevant current references. Any changes to the reference list should be mentioned in the rebuttal letter that accompanies your revised manuscript. If you need to cite a retracted article, indicate the article’s retracted status in the References list and also include a citation and full reference for the retraction notice.

[Note: HTML markup is below. Please do not edit.]

Reviewers' comments:

Reviewer's Responses to Questions

**Comments to the Author**

1. Is the manuscript technically sound, and do the data support the conclusions?

Reviewer #1: Yes

Reviewer #2: Partly

**********

2. Has the statistical analysis been performed appropriately and rigorously?

Reviewer #1: Yes

Reviewer #2: I Don't Know

**********

3. Have the authors made all data underlying the findings in their manuscript fully available?

The PLOS Data policy

Reviewer #1: No

Reviewer #2: No

**********

4. Is the manuscript presented in an intelligible fashion and written in standard English?

Reviewer #1: Yes

Reviewer #2: Yes

**********

Reviewer #1: SUMMARY

This study uses administrative claims data from the Iran Health Insurance Organization (IHIO) to analyze inpatient costs, revascularization rates, and length of stay (LOS) among 158,584 hospitalizations involving invasive coronary angiography (ICA) over a one-year period. Three-tier generalized linear mixed models (GLMMs) are used to examine patient-, hospital-, and province-level determinants of these outcomes. Weighted quantile sum (WQS) regression is additionally used to assess multi-pollutant effects on acute ICA rates. The paper addresses a genuine evidence gap in LMIC cardiovascular health economics. However, the authors should address the following concerns:

COMMENTS

1. (lines 83) The sentence "Data from 158,584 hospitalizations was analyzed" should be moved from the Results section to the Methods section of the abstract, as it describes the analytic sample rather than a study result.

2. (lines 88–89) The procedure complexity cost ratio reported in the abstract and results text (CR: 4.36; 95% CI 4.43–4.39) is inconsistent with Figure 3, which shows CR: 4.37 [4.34–4.39] for the total cohort. The text values appear to be a transcription error and must be corrected to match the figure.

3. Table 1 reports 1,690 in-hospital deaths (1.07%). The authors do not clarify how fatal hospitalizations were handled in the log-normal LOS and cost models. Given the small proportion, the practical impact is likely limited, but a brief statement clarifying whether these cases were included or excluded, and if included, why competing risks were not considered.

4. (lines 176–179) Provincial socioeconomic deprivation is derived from reference [20] but its construction is not described in sufficient detail for a standalone reading of the methods. Additionally, treating deprivation as a rank-ordered continuous variable in regression assumes proportional spacing between ranks, which may not hold and is not discussed.

5. The authors should clarify whether staged revascularization hospitalizations for the same patient (20.84% of revascularizations; n=15,598; Table 2) were included as independent observations in the models. If so, within-patient correlation across admissions is unaddressed, as section 2.5.4 states that only hospital and province IDs were treated as random intercepts, with no patient-level random intercept specified. The authors should therefor add a statement clarifying how staged hospitalizations were handled, or a sensitivity analysis restricted to index hospitalizations.

6. (lines 233–234, 405–408) The authors report ICC point estimates for province- and hospital-level variance components without accompanying CIs. Given the relatively small number of province-level clusters (N=31), reporting bootstrapped CIs around ICC estimates would help to better assess the precision and reliability of the reported variance partitioning.

7. The authors state that VIF tests confirmed no multicollinearity (line 236, lines 407–408) but do not report the actual VIF values anywhere in the manuscript. These should be reported, at minimum in supplementary materials, so that the claim of model stability can be independently verified.

8. (line 102) "progresses in mitigation" should read "progress in mitigation.

9. (line 430) "this observations" should read "these observations."

Reviewer #2: This research study is interesting and provides important information about ICA and related expenditures in Iran. However, the primary research question, or why, is a bit lost in all the complex analyses and results. It is difficult to interpret what specific research question each analysis is designed to answer. Additional rationale in the Methods would help in interpretation of results.

Please include the ICD-10 codes used for cohort identification and analysis in the supplementary materials. Further detail on the handling of missing data is needed - were missing values imputed, excluded, etc.

The results are dense and the interpretation is hard to follow or put into context. Reminding the reader what the intent of each statistical analysis is and what it is designed to answer would be very helpful.

**********

what does this mean?). If published, this will include your full peer review and any attached files.

If you choose “no”, your identity will remain anonymous but your review may still be made public.

**Do you want your identity to be public for this peer review?**  For information about this choice, including consent withdrawal, please see our Privacy Policy

Reviewer #1: No

Reviewer #2: No

**********

[NOTE: If reviewer comments were submitted as an attachment file, they will be attached to this email and accessible via the submission site. Please log into your account, locate the manuscript record, and check for the action link "View Attachments". If this link does not appear, there are no attachment files.]

To ensure your figures meet our technical requirements, please review our figure guidelines: https://journals.plos.org/plosone/s/figures

You may also use PLOS’s free figure tool, NAAS, to help you prepare publication quality figures: https://journals.plos.org/plosone/s/figures#loc-tools-for-figure-preparation.

NAAS will assess whether your figures meet our technical requirements by comparing each figure against our figure specifications.

# Author comment: Author response to Decision Letter 1

**DOI:** 10.1371/journal.pone.0356496.r002

4 Jul 2026

POINT-BY-POINT RESPONSE FORM

Manuscript #: PONE-D-26-16623

Manuscript title: Economic drivers and systemic disparities in invasive coronary angiography: Insights from a national claims database

We would like to thank the editor and reviewers for their invaluable comments to help improve our research article, and the time you devoted to reviewing this paper is of great value to us. Your comments have been carefully considered and meticulously applied to address the mentioned concerns/suggestions. We hope that the revised version meets your expectations.

Suggestion, Question,

or Comment from the

Editor and Journal Requirements Author’s Response Change in the Manuscript (*line numbers refer to the marked manuscript copy”)

1. Please ensure that your manuscript meets PLOS ONE's style requirements, including those for file naming. The PLOS ONE style templates can be found at

https://journals.plos.org/plosone/s/file?id=wjVg/PLOSOne_formatting_sample_main_body.pdf and

https://journals.plos.org/plosone/s/file?id=ba62/PLOSOne_formatting_sample_title_authors_affiliations.pdf

Thanks for your comment. We formatted the manuscript according to the mentioned guidelines.

2. Your ethics statement should only appear in the Methods section of your manuscript. If your ethics statement is written in any section besides the Methods, please delete it from any other section.

We appreciate you pointing this out. We removed the "Informed consent and ethical approval" subsection from the "Statements and Declarations" section at the end of the manuscript. The ethical statement is now exclusively located within the "2.6. Ethical considerations" subsection of the Methods section. We deleted the text from the "Statements and Declarations" section at the end of the manuscript:

Informed consent and ethical approval: This study was reviewed and approved by Research Ethics Committee of School of Public Health - Tehran University of Medical Sciences with the Approval ID: IR.TUMS.SPH.REC.1403.319; Approval Date: March 16, 2025. As this was a retrospective, cross-sectional analysis utilizing a fully de-identified national dataset, the research was classified as exempt from individual informed consent.

3. In the online submission form, you indicated that the data that support the findings of this study are held by the Iran Health Insurance Organization (IHIO) and are subject to legal and national security restrictions. The authors were granted access under a specific research agreement and are not legally permitted to share the data publicly or with third parties. Data access requests can be directed to the Research Committee of the Iran Health Insurance Organization (https://nchir-r.ihio.gov.ir/general/homePage.action, Email: intl@ihio.gov.ir).

All PLOS journals now require all data underlying the findings described in their manuscript to be freely available to other researchers, either 1. In a public repository, 2. Within the manuscript itself, or 3. Uploaded as supplementary information.

This policy applies to all data except where public deposition would breach compliance with the protocol approved by your research ethics board. If your data cannot be made publicly available for ethical or legal reasons (e.g., public availability would compromise patient privacy), please explain your reasons on resubmission and your exemption request will be escalated for approval. We sincerely appreciate and fully support the journal's commitment to open science and data transparency. However, we must respectfully request an exemption from the public data deposition requirement for this manuscript due to strict legal restrictions imposed by the data owner (IHIO).

The dataset utilized in our study comprises sensitive national administrative claims data encompassing 158,584 hospitalizations. Under the terms of our specific research agreement and the overarching policies of the Iran Health Insurance Organization (IHIO), public deposition of this data is strictly prohibited ,even in de-identified or anonymized formats, to safeguard patient privacy and comply with national data security regulations. The authors are legally prohibited from distributing this data publicly or to third parties.

To ensure that our research remains as transparent and reproducible as possible within these legal confines, we have provided the direct contact information for the IHIO Research Committee. Independent researchers who meet the criteria for accessing confidential health data may apply for access directly through them. We hope this explanation provides the necessary context for the editorial board to approve our exemption request.

Contact for Data Requests: Research Department of the Iran Health Insurance Organization (IHIO)

Website: https://nchir-r.ihio.gov.ir/general/homePage.action

Email: intl@ihio.gov.ir

Address: No.1 Derakhshan, N Falamak St., Eivanak Blvd, Qods (West) Town, Tehran. Iran

4. Please include captions for your Supporting Information files at the end of your manuscript, and update any in- text citations to match accordingly. Please see our Supporting Information guidelines for more information: http://journals.plos.org/plosone/s/supporting-information

Done.

5. If the reviewer comments include a recommendation to cite specific previously published works, please review and evaluate these publications to determine whether they are relevant and should be cited. There is no requirement to cite these works unless the editor has indicated otherwise. Properly noted.

6. Please review your reference list to ensure that it is complete and correct. If you have cited papers that have been retracted, please include the rationale for doing so in the manuscript text, or remove these references and replace them with relevant current references. Any changes to the reference list should be mentioned in the rebuttal letter that accompanies your revised manuscript. If you need to cite a retracted article, indicate the article’s retracted status in the References list and also include a citation and full reference for the retraction notice. We have conducted a thorough review of our reference list to ensure all citations are complete, accurate, and up to date. Thus, the reference list remains unchanged and all citations accurately support evidence presented in the manuscript.

Suggestion, Question,

or Comment from the

Reviewer 1 Author’s Response Change in the Manuscript (*line numbers refer to the marked manuscript copy”)

This study uses administrative claims data from the Iran Health Insurance Organization (IHIO) to analyze inpatient costs, revascularization rates, and length of stay (LOS) among 158,584 hospitalizations involving invasive coronary angiography (ICA) over a one-year period. Three-tier generalized linear mixed models (GLMMs) are used to examine patient-, hospital-, and province-level determinants of these outcomes. Weighted quantile sum (WQS) regression is additionally used to assess multi-pollutant effects on acute ICA rates. The paper addresses a genuine

evidence gap in LMIC cardiovascular health economics. However, the authors should address the following concerns: Many thanks for your encouraging comments. We are pleased to have satisfied your expectations of this manuscript.

1. (lines 83) The sentence "Data from 158,584 hospitalizations was analyzed" should be moved from the Results section to the Methods section of the abstract, as it describes the analytic sample rather than a study result. Done. Page 2, line 232

2. (lines 88–89) The procedure complexity cost ratio reported in the abstract and results text (CR: 4.36; 95% CI 4.43–4.39) is inconsistent with Figure 3, which shows CR: 4.37 [4.34–4.39] for the total cohort. The text values appear to be a transcription error and must be corrected to match the figure. Thanks for your comment. Done. Page 2, lines 241

3. Table 1 reports 1,690 in-hospital deaths (1.07%). The authors do not clarify how fatal hospitalizations were handled in the log-normal LOS and cost models. Given the small proportion, the practical impact is likely limited, but a brief statement clarifying whether these cases were included or excluded, and if included, why competing risks were not considered. We thank you for this observation and for allowing us the opportunity to clarify our methodological approach regarding this patient subset.

Fatal hospitalizations were included in all log-normal LOS and cost models. From a health economics and health systems perspective, it is important to capture the complete financial and resource utilization burden borne by the payer. Patients who die during their hospital stay often require high-intensity, high-cost care prior to death; excluding them would artificially underestimate the true systemic economic burden of acute and complex coronary presentations.

In our study total inpatient cost and total LOS were evaluated as realized, continuous variables representing cumulative resource utilization, modeled via log-normal generalized linear mixed models (GLMMs), rather than time-to-event probabilities. As you accurately noted, the small proportion of these cases inherently limits their practical impact on the systemic estimates, but their inclusion ensures the integrity of the total economic footprint. We have added a brief clarification to the Methods section to ensure this is transparent to the readership. Page 9, lines 407-409

4. (lines 176–179) Provincial socioeconomic deprivation is derived from reference [20] but its construction is not described in sufficient detail for a standalone reading of the methods. Additionally, treating deprivation as a rank- ordered continuous variable in regression assumes proportional spacing between ranks, which may not hold and is not discussed. We appreciate your comment about the deprivation index. We have expanded our description of the deprivation index to clarify that it is based on a systematic review of multideprivation patterns across 26 studies in Iran, encompassing domains such as housing, employment, and infrastructure.

Regarding the index in the regression models: you raise a very valid point. Treating a rank-ordered variable as continuous does assume proportional spacing, which is often violated in composite development indices. However, treating a 31-level rank variable categorically would over-parameterize our provincial-level model (N=31). To address this, we maintained it as a continuous rank but acknowledged this methodological limitation - and its potential to mask non-linear threshold effects - in the Discussion section. Page 6, lines 354-356

Page 25, lines 830-832

5. The authors should clarify whether staged revascularization hospitalizations for the same patient (20.84% of revascularizations; n=15,598; Table 2) were included as independent observations in the models. If so, within- patient correlation across admissions is unaddressed, as section 2.5.4 states that only hospital and province IDs were treated as random intercepts, with no patient-level random intercept specified. The authors should therefor add a statement clarifying how staged hospitalizations were handled, or a sensitivity analysis restricted to index hospitalizations.

Thanks for your careful attention to the independence of observations in our models.

Staged revascularization hospitalizations (representing 20.84% of revascularizations; n = 15,598) were not included as independent observations in our models. Instead, when a patient required a planned, staged revascularization during a subsequent admission, the procedural data, associated costs, and length of stay (LOS) from that subsequent admission were fully aggregated into that patient's single index hospitalization record. Therefore, each care episode represents a single, comprehensive observation per patient in our modeling framework.

Because these staged events were consolidated into a single index record rather than treated as separate entries, within-patient correlation across multiple rows of data does not apply to our multilevel models. To prevent model singularity and overfitting, we focused on hospital and province IDs as random intercepts, which perfectly aligns with our data structure since the overwhelming majority of the cohort (94.1%) contributed a single, unified care record. We agree that this aggregation strategy is vital for understanding our predictive models, and we have updated the Methods section to ensure full transparency. Page 7, lines 366-368

6. (lines 233–234, 405–408) The authors report ICC point estimates for province- and hospital-level variance components without accompanying CIs. Given the relatively small number of province-level clusters (N=31), reporting bootstrapped CIs around ICC estimates would help to better assess the precision and reliability of the reported variance partitioning. Thanks for your meticulous and valuable comment. We added the 95%CI to the text. Page 9, lines 417-419

Page 20, lines 703-705

7. The authors state that VIF tests confirmed no multicollinearity (line 236, lines 407–408) but do not report the actual VIF values anywhere in the manuscript. These should be reported, at minimum in supplementary materials, so that the claim of model stability can be independently verified. We sincerely thank you for your recommendation. We added a table in the supplementary file for this. S6 Table in the supplementary file

8. (line 102) "progresses in mitigation" should read "progress in mitigation. Done. Page 3, lines 269

9. (line 430) "this observations" should read "these observations." Done. Page 21, lines 738

Suggestion, Question,

or Comment from the

Reviewer 2 Author’s Response Change in the Manuscript (*line numbers refer to the marked manuscript copy”)

This research study is interesting and provides important information about ICA and related expenditures in Iran. We sincerely thank you for your positive assessment of our study's scope.

However, the primary research question, or why, is a bit lost in all the complex analyses and results. It is difficult to interpret what specific research question each analysis is designed to answer. Additional rationale in the Methods would help in interpretation of results. Thank you for highlighting this important point. We added the rationale in the methods section. Page 10, line 422-428

Please include the ICD-10 codes used for cohort identification and analysis in the supplementary materials. We appreciate your comment regarding diagnostic definitions. We added a supplementary table for ICD-10 coding definitions used for clinical classification. S1 Table in the supplementary file

Further detail on the handling of missing data is needed - were missing values imputed, excluded, etc. We appreciate you pointing out the need for greater clarity regarding our missing data protocols.

For patient-level data (demographics, clinical diagnoses, and billing codes), missingness was exceptionally low. This is because administrative claims databases mandate the completion of core demographic and diagnostic fields for a hospitalization to be successfully billed and recorded. Therefore, hospitalizations with any missing essential demographic or clinical data were simply excluded from the cohort to maintain a complete-case analysis for the hierarchical models.

Conversely, for the meteorological and air quality datasets, missing values are more common due to routine sensor downtime or calibration periods. For these environmental variables, missing values were imputed using Multivariate Imputation by Chained Equations (MICE) prior to lag calculations. We have updated the Methods section to make this dual approach completely transparent to the reader. Page 5, line 328-329

Page 7, line 372

Page 8, line 385

The results are dense and the interpretation is hard to follow or put into context. Reminding the reader what the intent of each statistical analysis is and what it is designed to answer would be very helpful. Thanks for your careful attention to this matter again, we have already clarified the intent of each statistical analysis in the methods section based on your previous comment. Page 10, line 422-428

Attachment Submitted filename: Response to Reviewers.docx

# Peer review: Decision Letter 1

**DOI:** 10.1371/journal.pone.0356496.r003

**Authors:** Hossein Ali Adineh

4 Aug 2026

Economic drivers and systemic disparities in invasive coronary angiography: Insights from a national claims database

PONE-D-26-16623R1

Dear Dr. Semnani,

We’re pleased to inform you that your manuscript has been judged scientifically suitable for publication and will be formally accepted for publication once it meets all outstanding technical requirements.

Within one week, you’ll receive an e-mail detailing the required amendments. When these have been addressed, you’ll receive a formal acceptance letter and your manuscript will be scheduled for publication.

An invoice will be generated when your article is formally accepted. Please note, if your institution has a publishing partnership with PLOS and your article meets the relevant criteria, all or part of your publication costs will be covered. Please make sure your user information is up-to-date by logging into Editorial Manager at Editorial Manager® and clicking the ‘Update My Information' link at the top of the page. For questions related to billing, please contact billing support.

If your institution or institutions have a press office, please notify them about your upcoming paper to help maximize its impact. If they’ll be preparing press materials, please inform our press team as soon as possible -- no later than 48 hours after receiving the formal acceptance. Your manuscript will remain under strict press embargo until 2 pm Eastern Time on the date of publication. For more information, please contact onepress@plos.org.

Kind regards,

Hossein Ali Adineh, Ph.D

Academic Editor

PLOS One

Additional Editor Comments (optional):

Reviewers' comments:

Reviewer's Responses to Questions

**Comments to the Author**

Reviewer #1: All comments have been addressed

Reviewer #2: All comments have been addressed

**********

2. Is the manuscript technically sound, and do the data support the conclusions?

Reviewer #1: Yes

Reviewer #2: Yes

**********

3. Has the statistical analysis been performed appropriately and rigorously?

Reviewer #1: Yes

Reviewer #2: Yes

**********

4. Have the authors made all data underlying the findings in their manuscript fully available?

The PLOS Data policy

Reviewer #1: No

Reviewer #2: Yes

**********

5. Is the manuscript presented in an intelligible fashion and written in standard English?

Reviewer #1: Yes

Reviewer #2: Yes

**********

Reviewer #1: (No Response)

Reviewer #2: The authors have adequately addressed my comments. I have no additional comments or edits at this time.

**********

what does this mean?). If published, this will include your full peer review and any attached files.

If you choose “no”, your identity will remain anonymous but your review may still be made public.

**Do you want your identity to be public for this peer review?**  For information about this choice, including consent withdrawal, please see our Privacy Policy

Reviewer #1: No

Reviewer #2: **Yes:** Kael Wherry

**********

# Editor report: Acceptance letter

**DOI:** 10.1371/journal.pone.0356496.r004

**Authors:** Hossein Ali Adineh

PONE-D-26-16623R1

PLOS One

Dear Dr. Semnani,

I'm pleased to inform you that your manuscript has been deemed suitable for publication in PLOS One. Congratulations! Your manuscript is now being handed over to our production team.

At this stage, our production department will prepare your paper for publication. This includes ensuring the following:

* All references, tables, and figures are properly cited

* All relevant supporting information is included in the manuscript submission,

* There are no issues that prevent the paper from being properly typeset

You will receive further instructions from the production team, including instructions on how to review your proof when it is ready. Please keep in mind that we are working through a large volume of accepted articles, so please give us a few days to review your paper and let you know the next and final steps.

Lastly, if your institution or institutions have a press office, please let them know about your upcoming paper now to help maximize its impact. If they'll be preparing press materials, please inform our press team within the next 48 hours. Your manuscript will remain under strict press embargo until 2 pm Eastern Time on the date of publication. For more information, please contact onepress@plos.org.

You will receive an invoice from PLOS for your publication fee after your manuscript has reached the completed accept phase. If you receive an email requesting payment before acceptance or for any other service, this may be a phishing scheme. Learn how to identify phishing emails and protect your accounts at https://explore.plos.org/phishing.

If we can help with anything else, please email us at customercare@plos.org.

Thank you for submitting your work to PLOS One and supporting open access.

Kind regards,

PLOS ONE Editorial Office Staff

on behalf of

Dr. Hossein Ali Adineh

Academic Editor

PLOS One
